# GCN2 deficiency protects mice from denervation-induced skeletal muscle atrophy via inhibiting FoxO3a nuclear translocation

**DOI:** 10.1007/s13238-018-0504-0

**Published:** 2018-01-18

**Authors:** Yuting Guo, Huiwen Wang, Yinglong Tang, Yue Wang, Mengqi Zhang, Zhiguang Yang, Eric Nyirimigabo, Bin Wei, Zhongbing Lu, Guangju Ji

**Affiliations:** 1 College of Life Science, University of Chinese Academy of Sciences, Beijing, 100049 China; 2 National Laboratory of Biomacromolecules, Institute of Biophysics, Chinese Academy of Sciences, Beijing, 100101 China

**Dear Editor**,

Several recent clinical studies have indicated that dietary supplementation with branched-chain amino acids (BCAA), particularly with leucine, is an effective anti-atrophic therapy (Bauer et al., 2015; Tsien et al., 2015; English et al., 2016). In animal models, BCAA can prevent denervation (Ribeiro et al., 2015), hindlimb suspension (Maki et al., 2012; Jang et al., 2015) or dexamethasone-induced (Yamamoto et al., 2010) muscle atrophy. General control nonderepressible 2 kinase (GCN2) is a well-known amino-acid sensor. Under conditions of amino-acid deprivation, the increased level of uncharged transfer RNA (tRNA) activates GCN2 through binding to the histadyl-tRNA synthetase-like domain (Wek et al., 1995). Upon activation, GCN2 phosphorylates eukaryotic initiation factor 2 alpha at Ser51, which leads to translational arrest and restoration of amino acid homeostasis (Wek et al., 1995; Sood et al., 2000). As amino acids are potent modulators of protein turnover in skeletal muscle, we proposed that GCN2 may affect denervation-induced muscle atrophy, but the detail mechanism remains unclear.

To investigate the impact of GCN2 on the development of muscle atrophy, we performed sciatic denervation procedure on hindlimb muscles in wild-type (WT) and *Gcn2*^−/−^ mice. On day 7 after denervation, WT tibial anterior (TA) muscle mass decreased to 70.9% ± 1.8% of the contralateral level, while GCN2-deficient TA muscle mass remained at 83.1% ± 1.6% of its contralateral level (Fig. 1A). GCN2 deficiency also significantly attenuated the muscle mass loss in atrophied gastrocnemius (GAS) and extensor digitorum longus (EDL) muscles. Similar results were observed on day 14 after denervation (Fig. S1). Wheat germ agglutinin (WGA) staining of muscle cryosections demonstrated that GCN2-deficient TA muscles had a better preservation of myofiber size in response to denervation (Fig. 1B). After denervation, myofiber size distribution calculated from WT TA muscles showed a leftward shift from its contralateral conditions. However, such shift was delayed in atrophied GCN2-deficient TA muscles (Fig. 1C). To further verify whether activation of GCN2 contributes to muscle atrophy, we overexpressed GCN2 in flexor digitorum brevis (FDB) muscles using *in vivo* electroporation. The transfection efficiency was confirmed by Western blot (Fig. S2). After denervation for ten days, the diameter of WT FDB myofiber decreased from 37.2 ± 0.9 μm to 24.2 ± 0.7 μm, while the diameter of *Gcn2*^−/−^ FDB myofiber was maintained at 32.3 ± 0.7 μm. Overexpression of GCN2 resulted in a further reduction in the diameter of FDB myofibers in both WT and *Gcn2*^−/−^ mice. However, the reduction was more dramatically in *Gcn2*^−/−^ FDB muscle and the significant difference in the diameter of atrophied FDB myofiber between WT and *Gcn2*^−/−^ mice was diminished after transfected with the GCN2 plasmid (Fig. 1D).

**Figure 1:**
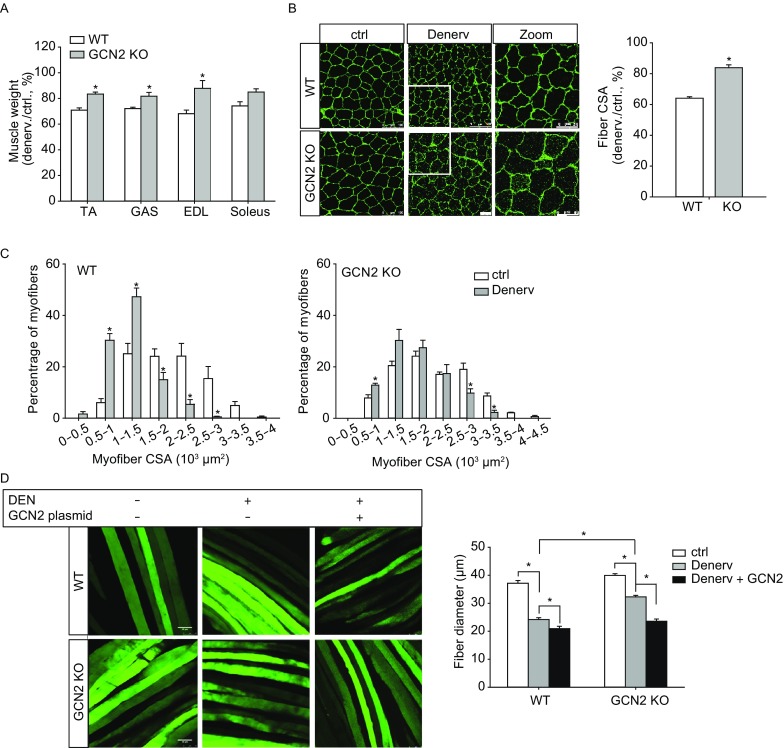
**Effect of GCN2 on denervation-induced atrophy**. Sciatic denervation was performed on 2- to 3-month-old WT and *Gcn2*^−/−^ mice. (A) Seven days after denervation, the percentage of denervated to contralateral skeletal muscle mass was determined. ctrl, contralateral; denerv, denervated. (B) Cryosections from contralateral and denervated TA muscle of WT and *Gcn2*^−/−^ mice were stained with wheat germ agglutinin (WGA). Scale bars = 100 μm. The average myofiber cross-sectional area (CSA) was measured by Image J, and data are expressed as the percentage of denervated to contralateral fiber CSA of WT and *Gcn2*^−/−^ mice. (C) TA myofiber size distribution showed a reduced leftward shift in GCN2-deficient TA muscles compared to WT muscles. (D) Flexor digitorum brevis (FDB) muscle fibers were transfected with the pIRS2-EGFP or pIRS2-EGFP-GCN2 plasmid. On day 10 after denervation, representative images were taken and FDB myofiber diameters were measured. 4 mice were used for each group and 20–40 myofiber diameters were measured for each mouse. Scale Bar = 50 μm. *P < 0.05 compared to WT or control mice

Emerging evidence suggests that the protein degradation in muscle atrophy is mediated by FoxO3a, an important regulator of *Atrogin*-*1*, *MuRF*-*1* and *LC3* (Sandri et al., 2004; Zhao et al., 2007; Guo et al., 2016). Upon atrophy stimuli, FoxO3a shuffled into the myofiber nucleus, which leads to transcriptional activation (Sandri et al., 2004). Through Western blotting and immunostaining, we observed that the phosphorylation level of FoxO3a at Ser207 were increased in TA muscles of WT mice after denervation, and FoxO3 was mainly located in the nucleus. However, the denervation-induced phosphorylation and nuclear accumulation of FoxO3a were significantly less in GCN2-deficient muscles compared with that in WT muscles (Fig. 2A–B). We also observed significant increases in protein expression of MuRF-1 and Atrogin-1, as well as the ratio of conversion of LC3 into the activated forms (LC3-II/I) in WT atrophic TA muscles. However, increases in E3 ubiquitin ligases expression and activation of autophagy were statistically less remarkable in atrophic *Gcn2*^−/−^ muscles than in WT (Fig. S3).

**Figure 2:**
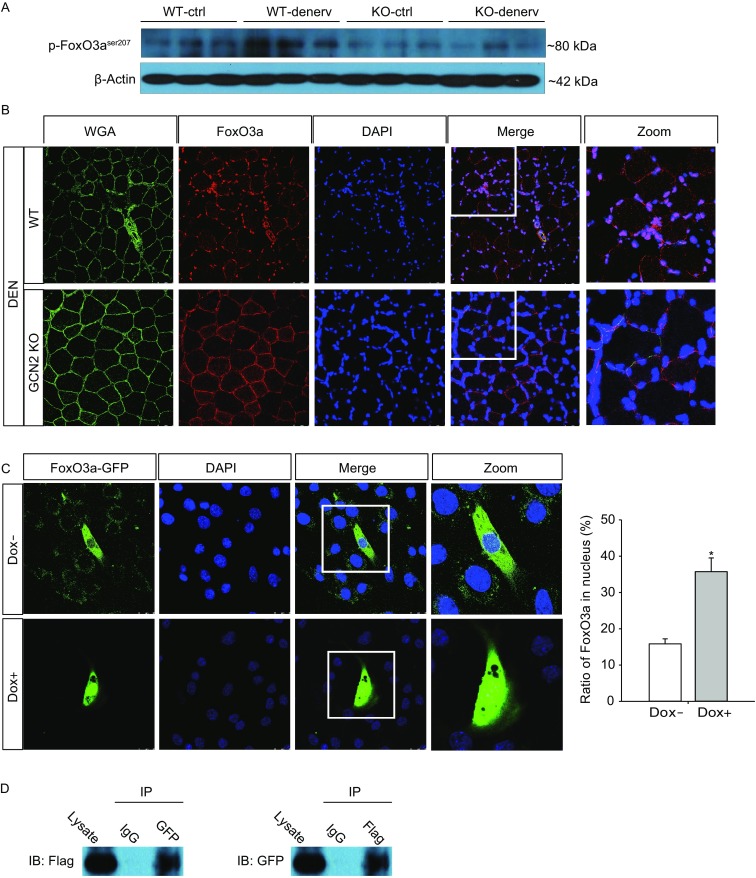
**Effect of GCN2 on FoxO3a activation and nuclear translocation**. (A) TA muscles were collected from WT and GCN2^−/−^ mice seven days after denervation, and lysates were examined by Western blotting. The immunoblot band intensities were quantified by densitometry and the normalized values with respect to corresponding loading controls (β-actin) are plotted as bar graphs in panels. (B) Representative immunostaining using WGA and FoxO3a antibody on TA muscle sections obtained on denervation day 7. (C) C2C12 cells were stably transfected with doxycycline (Dox)-controlled Flag-tagged mouse GCN2 (mGCN2-C2C12), cultured for 24 h with (Dox+) or without (Dox−) 1 μg/mL Dox, and analyzed for expression of Flag-tagged GCN2 by Western blotting. (D) mGCN2-C2C12 cells were transfected with pEGFP-C2-FoxO3a plasmid, cultured for 48 h with (Dox+) or without (Dox−) 1 μg/mL Dox. Representative fluorescent images were taken and the ratio of FoxO3a in the nucleus was quantitatively analyzed. (D) mGCN2-C2C12 cells were transfected with pEGFP-C2-FoxO3a plasmid and grown for 48 h with 1 μg/mL Dox. Anti-GFP and anti-Flag antibodies were used to immunoprecipitate (IP) GFP-FoxO3a and Flag-GCN2 from whole-cell extracts, respectively, followed by Western blotting analyses using anti-Flag (left) and anti-GFP (right) antibodies to detect Flag-GCN2 and GFP-FoxO3a, respectively. Figures are chosen as the representative of three independent experiments. *P < 0.05

To investigate whether GCN2 activation directly causes FoxO3a nuclear translocation in muscle atrophy, we generated a stable C2C12 cell line (mGCN2-C2C12) with doxycycline (Dox)-controlled expression of flag-tagged mouse GCN2 (Fig. 2C), and found that GCN2 overexpression induced by Dox (Dox+) significantly increased the ratio of FoxO3a in the nucleus (Fig. 2D). Furthermore, co-immunoprecipitation experiments using lysates from FoxO3a plasmid-transfected mGCN2-C2C12 cells with Dox demonstrated that GCN2-Flag and FoxO3a-EGFP were specifically co-precipitated with anti-GFP and anti-Flag antibodies, respectively (Fig. 2E), demonstrating that GCN2 and FoxO3a can physically interact with each other in cells. Using the differentiated mGCN2-C2C12 cells, we also found that overexpression of GCN2 exacerbated dexamethasone-induced upregulation of Atrogin-1 and LC3-II in differentiated C2C12 cells (Fig. S4).

It has been demonstrated dietary deprivation of essential amino acids, which activates GCN2 via increasing uncharged tRNA levels (Wek et al., 1995), caused diffuse atrophy in the rectus femoris muscles (Kamata et al., 2014). In contrast, leucine or other BCAA supplementation, which attenuates GCN2 activity (Wek et al., 1995), has been regarded as a potential pharmaconutrient for the treatment of numerous muscle wasting conditions (Bauer et al., 2015; Tsien et al., 2015; English et al., 2016). In agreement with those findings, we demonstrated that GCN2 deletion attenuates, whereas GCN2 overexpression exacerbates denervation-induced muscle atrophy. Furthermore, the detrimental effect of GCN2 in denervation-induced atrophy was related to FoxO3a activation, which upregulates genes involved in both the ubiquitin-proteasome pathway and autophagy in muscle atrophy (Sandri et al., 2004; Bertaggia et al., 2012; Wei et al., 2013; Guo et al., 2016). Thus, reducing GCN2 activity may be a potential therapeutic approach for the clinical treatment of muscle atrophy.

## FOOTNOTES

This study was supported by grants from the National Natural Science Foundation of China (Grant Nos. 81470520, 91643206, 91743104, 31371430 to HW and 31300976 to BW) and Chinese Academy of Sciences (KJRH2015-005 and Hundred Talents Program).

Yuting Guo, Huiwen Wang, Yinglong Tang, Yue Wang, Mengqi Zhang, Zhiguang Yang, Eric Nyirimigabo, Bin Wei, Zhongbing Lu, and Guangju Ji declare that they have no conflicts of interest. All institutional and national guidelines for the care and use of laboratory animals were followed.

## Electronic supplementary material

Below is the link to the electronic supplementary material.

Supplementary material 1 (PDF 474 kb)
