# Supplementary material for: GCN2 deficiency protects mice from denervation-induced skeletal muscle atrophy via inhibiting FoxO3a nuclear translocation
Source: Protein Cell. 2018 Jan 18;9(11):966–70. doi: 10.1007/s13238-018-0504-0 (PMC6208481; doi:10.1007/s13238-018-0504-0)
Supplement: Supplementary file 1 — Supplementary material 1 (PDF 474 kb) [file 13238_2018_504_MOESM1_ESM.pdf]

Supplemental Files

GCN2 deficiency protects mice from denervation-induced skeletal muscle atrophy via inhibiting FoxO3a nuclear translocation

Yuting Guo[1, 2†], Huiwen Wang[2†], Yinglong Tang[2†], Yue Wang[1], Mengqi Zhang[2], Zhiguang Yang[2], Eric Nyirimigabo[2], Bin Wei[2], Zhongbing Lu[1*], Guangju Ji[2*]

[1]College of Life Science, University of Chinese Academy of Sciences, Beijing, 100049, China

[2]National Laboratory of Biomacromolecules, Institute of Biophysics, Chinese Academy of Sciences, Beijing, 100101, China

† These authors contributed equally.

* Corresponding authors:

Zhongbing Lu, Ph.D,

19A Yuquanlu, Beijing, 100049, China

Fax: 86-10-69672630; Tel: 86-10-69672630

E-mail: luzhongbing@ucas.ac.cn

or

Guangju Ji, PhD

Datun Road 15, Chaoyang district, Beijing, China, 100101

Fax: 86-10-64871293; Tel: 86-10-64846720

Gj28@ibp.ac.cn

Materials and Methods

Mice and Denervation-Induced Muscle Atrophy

Male C57BL/6J (HFK Bioscience Co., Beijing) and GCN2$^{-/-}$ mice (Harding et al., 2000) (congenic with the C57BL/6J strain, kindly provided by Dr Yingjie Chen from University of Minnesota), 8–10 weeks of age, were used. Animal studies were performed in accordance with the principles of laboratory animal care (NIH publication no. 85-23, revised 1985) and with approval by the University Of Chinese Academy of Science Animal Care and Use Committee. The denervation procedure was performed on WT (n=20) and GCN2$^{-/-}$ mice (n=23) as previously described (Wei et al., 2013; Tang et al., 2015). Muscle samples were harvested at 7 days or 14 day after the denervation surgery.

Cross-Sectional Area Assessment

As described previously(Wei et al., 2013), frozen muscle sections (8 μm) were stained wheat germ agglutinin (WGA) and the cross-sectional area (CSA) was quantified using NIH Image J software (Bethesda, Maryland, USA). At least 5 mice/group were used for these experiments. The results were expressed as the mean CSA ±S.E. and as the percentage of fibers distributed.

Electroporation of Flexor Digitorum Brevis (FDB) Muscle

In vivo transfection experiments were carried out on 8-week-old WT (n=12) and GCN2$^{-/-}$ (n=12) mice according to a published procedure (DiFranco et al., 2009).

Briefly, mice were first anaesthetized and received a single injection of 0.4 U of hyaluronidase (Sigma) in 25 μl PBS into the flexor digitorum brevis (FDB) muscle. After 1 h, 10μg of control plasmid pIRS2-EGFP or pIRS2-EGFP-GCN2 was injected into each FDB, followed by 20 electrical pulses with a duration of 20 ms to ensure the absorption of the plasmid. The electrical field intensity was 100 V/cm. Ten days after electroporation, mice were euthanized and muscles were collected.

Cell Culture

C2C12 myoblasts were obtained from the American Type Culture Collection (ATCC, Manassas, VA) and grown in DMEM supplemented with 10% FBS and 1% penicillin and streptomycin. Myoblast fusion and differentiation was induced in subconfluent cells by replacing the medium with DMEM supplemented with 2% horse serum. To generate a stable, doxycycline-inducible GCN2 overexpression cell line, 5×10^5 exponentially growing cells were transfected with pLVX-Tet3G-GCN2 lentivirus for 24 h, followed by puromycin selection (1μg/mL) for 3 weeks.

Immunoprecipitation

The mouse FoxO3a cDNA in the pEGFP-C2 vector was transfected into mGCN2-C2C12 cells using Lipofectamine 2000 (Invitrogen, Grand Island, NY, USA) according to the manufacturer's instruction. Doxycycline was added to the cells 12 h post-transfection to inducing GCN2 expression. After transfection for 48 h, the cells were collected and lysed with the EBC lysis buffer, which contained 50 mmol/L

Tris(pH8.0), 120 mmol/L NaCl, 0.5% NP-40 and protease inhibitor cocktail (Roche, Switzerland). Immunoprecipitation was performed as previously reported (Bi et al., 2010). Briefly, lysates were precleared with protein A/G Plus-agarose beads (Santa Cruz, Dallas, Texas, USA) at 4 ℃ for 20 min. Following the removal of the beads by centrifugation, lysates were incubated with anti-Flag or anti-GFP antibodies in the presence of 15 μl of protein A/G Plus-agarose beads overnight at 4 ℃. After washing four times, the immunoprecipitates were subjected to immunoblotting. At least 3 independent experiments were performed.

Western Blotting

Freshly isolated TA muscle (10-20 mg) was homogenized in buffer (50 mM Tris-Cl, 150mM NaCl, 100 μg/ml phenylmethylsulfonyl fluoride, protease and phosphatase inhibitor cocktail from Roche and 1%Triton X-100) on ice for 30 min. After centrifugation at 12,000 ×g for 20 min at 4 ℃, the supernatant was used for western blot analysis as previously described (Guo et al., 2016). Primary antibodies used in this study were as follows: the antibodies against GCN2 and LC-3 were from Cell Signaling Technology (Danvers, MA, USA); the antibodies against Flag, GFP and phospho-FoxO3a^{ser207} were from Invitrogen (Grand Island, NY, USA); and the antibodies against Atrogin-1, MuRF-1 and β-actin were from Abcam (Cambridge, UK).

Data and Statistical Analysis. All values are expressed as the mean ± standard error.

Statistical significance was defined as $p < 0.05$. One-way or two-way analysis of variance (ANOVA) was used to test each variable for differences among the treatment groups with StatView (SAS Institute Inc). If ANOVA demonstrated a significant effect, pair-wise post hoc comparisons were made with Fisher's least significant difference test.

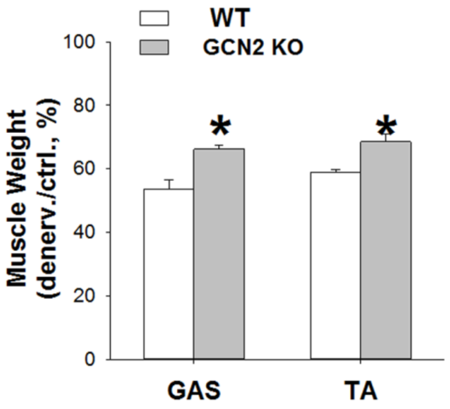

Supplemental Figure 1. GCN2 deficiency attenuated denervation induced muscle weight loss. Sciatic denervation was performed on 2-3 month old WT and GCN2$^{-/-}$ mice. Fourteen days after denervation, the percentage of denervated to contralateral skeletal muscle mass was determined. * $p<0.05$ compared to WT or control mice.

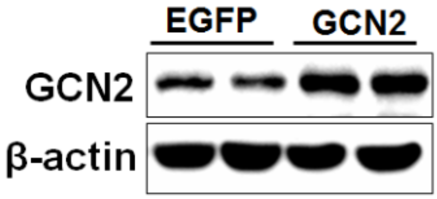

Supplemental Figure 2. In vivo electroporation of pIRS2-EGFP-GCN2 plasmid increased GCN2 expression in WT flexor digitorum brevis (FDB) muscles. Ten days after electroporation, FDB muscles were collected from WT mice and followed by western blot analysis.

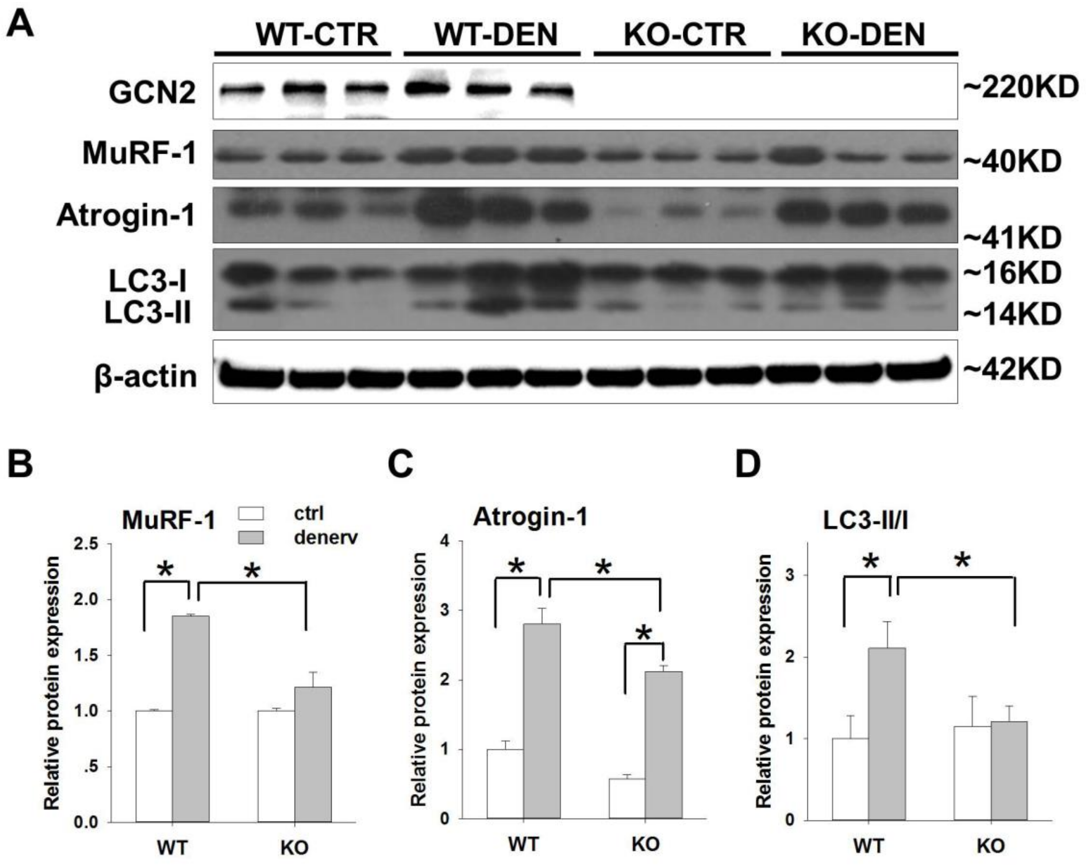

Supplemental Figure 3. Effect of GCN2 on expression of E3 ligases and autophagy in contralateral and denervated TA muscles. (A) Tissues were collected from WT and GCN2$^{-/-}$ mice seven days after denervation and lysates were examined by western blot. (B-C) The immunoblot band intensities were quantified by densitometry and the normalized values with respect to corresponding loading controls (β-actin) are plotted as bar graphs in panels. * $p<0.05$

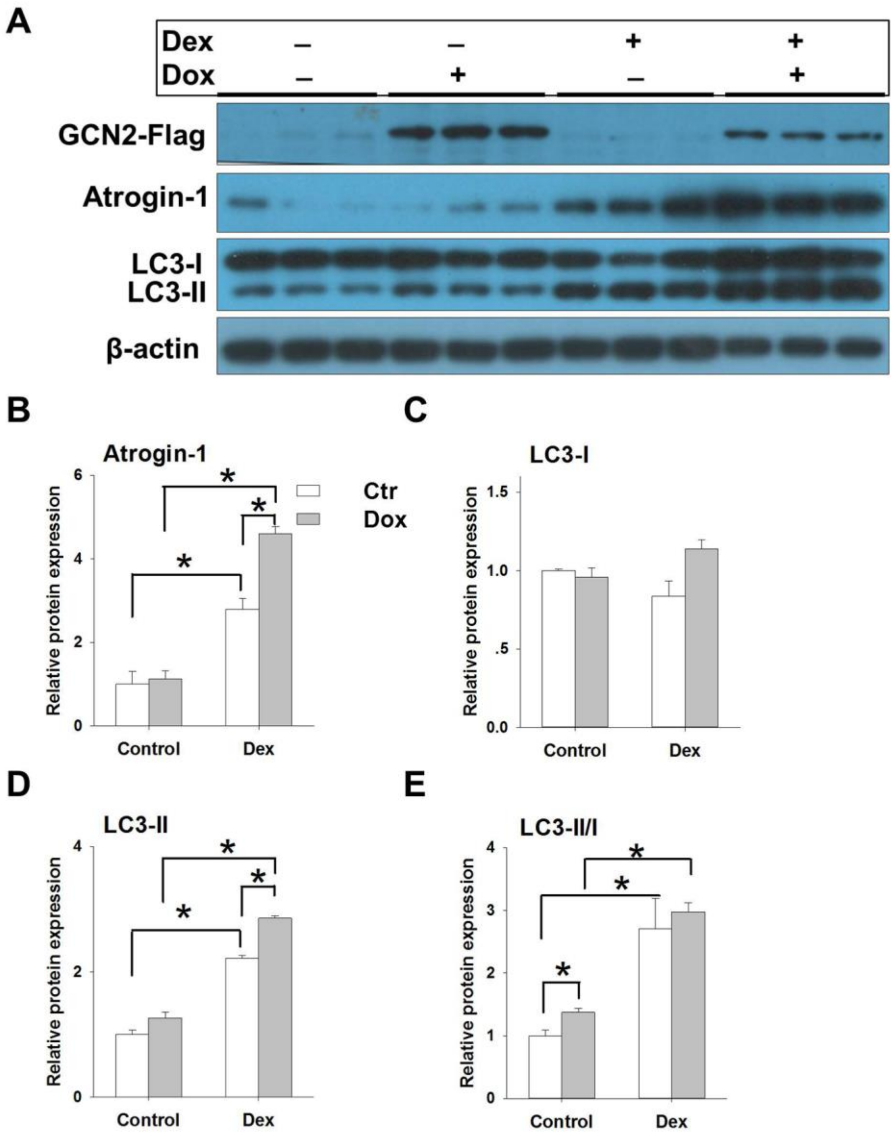

Supplemental Figure 4. Effect of GCN2 overexpression on protein degradation in control and dexamethasone treated C2C12 myotubes. mGCN2-C2C12 myoblasts were differentiated for 7days and then treated with or without 1 μg/ml of doxycycline (Dox) for 24 h. (A) Lysates were collected from control or dexamethasone (Dex) treated (1 μM, 6 h) myotubes and examined by western blot. (B-E) The immunoblot band intensities were quantified by densitometry and the normalized values with respect to corresponding loading controls (β-actin) are plotted as bar graphs in panels. * $p<0.05$

References

Bi, W., Xiao, L., Jia, Y., Wu, J., Xie, Q., Ren, J., Ji, G., and Yuan, Z. (2010). c-Jun N-terminal kinase enhances MST1-mediated pro-apoptotic signaling through phosphorylation at serine 82. J Biol Chem 285, 6259-6264.

DiFranco, M., Quinonez, M., Capote, J., and Vergara, J. (2009). DNA transfection of mammalian skeletal muscles using in vivo electroporation. J Vis Exp.

Guo, Y., Meng, J., Tang, Y., Wang, T., Wei, B., Feng, R., Gong, B., Wang, H., Ji, G., and Lu, Z. (2016). AMP-activated kinase alpha2 deficiency protects mice from denervation-induced skeletal muscle atrophy. Arch Biochem Biophys 600, 56-60.

Harding, H.P., Novoa, I., Zhang, Y., Zeng, H., Wek, R., Schapira, M., and Ron, D. (2000). Regulated translation initiation controls stress-induced gene expression in mammalian cells. Mol Cell 6, 1099-1108.

Tang, Y., Wang, H., Wei, B., Guo, Y., Gu, L., Yang, Z., Zhang, Q., Wu, Y., Yuan, Q., Zhao, G., *et al.* (2015). CUG-BP1 regulates RyR1 ASI alternative splicing in skeletal muscle atrophy. Sci Rep 5, 16083.

Wei, B., Dui, W., Liu, D., Xing, Y., Yuan, Z., and Ji, G. (2013). MST1, a key player, in enhancing fast skeletal muscle atrophy. BMC Biol 11, 12.
